# An Ecchordosis Physaliphora, a Rare Entity, Involving the Central Nervous System: A Systematic Review of the Literature

**DOI:** 10.3390/neurolint15040075

**Published:** 2023-09-26

**Authors:** Rajesh K. Gupta, Thejasvi A. Reddy, Ashutosh Gupta, Rohan Samant, Carlos A. Perez, Anam Haque

**Affiliations:** 1 Division of Multiple Sclerosis and Neuroimmunology, Department of Neurology, University of Texas Health Science Center, Houston, TX 77030, USA; 2 McGovern Medical School, UT Health Science Center, Houston, TX 77030, USAanam.haque@uth.tmc.edu (A.H.); 3 Department of Radiology, UT Health Science Center, Houston, TX 77030, USA; rohan.samant@uth.tmc.edu; 4 Department of Neurology, Baylor College of Medicine, Houston, TX 77030, USA; carlos.perez@bcm.edu

**Keywords:** ecchordosis physaliphora, notochord, chordoma, CNS

## Abstract

Ecchordosis physaliphora (EP) is a benign notochordal remnant, which is often an incidental finding; however, it can rarely present with neurological symptoms. We performed a systematic review of the literature for cases of symptomatic EP published in PubMed, Web of Science and Embase from January 1982 to May 2023. This is the largest review to date and revealed 60 cases including ours. Headache (55%) and CSF rhinorrhea (32%) were the most frequent clinical manifestations. The majority of symptomatic EP lesions were located in the prepontine region (77%) and required surgical resection (75%). EP should be considered in patients with neurologic symptoms in the setting of prepontine or posterior sphenoid sinus lesions. While symptomatic patients often require surgical intervention, rare cases may respond to oral corticosteroids.

## 1. Introduction

**Figure 1:**
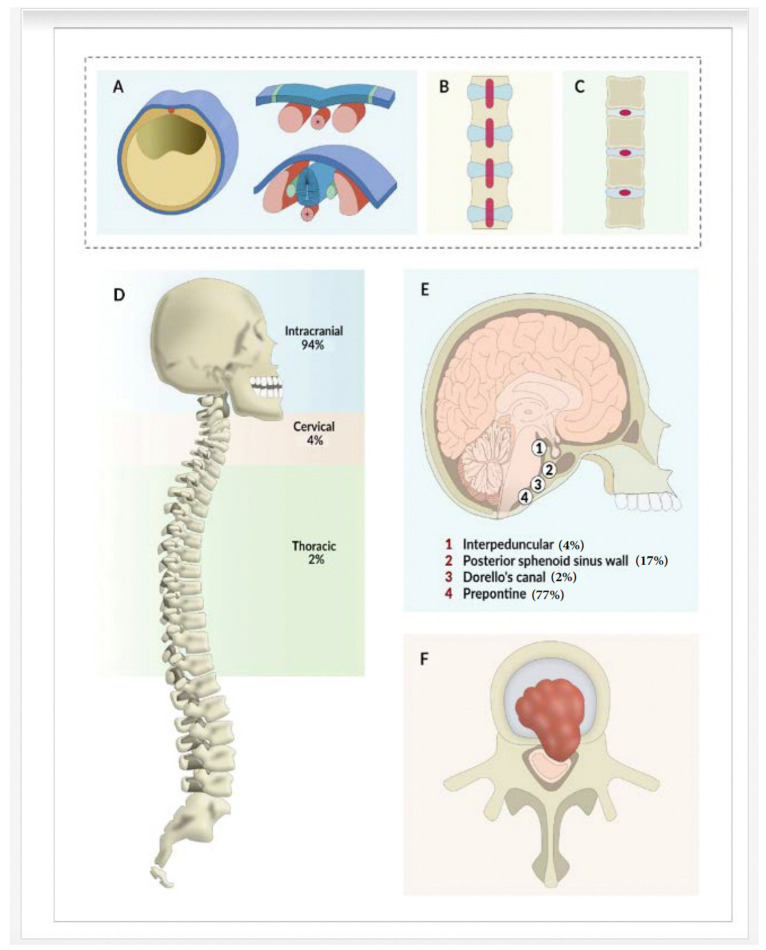
Development of EP from notochord and common locations. (**A**–**C**) showing notochordal development of EP, (**D**–**F**) showing location with percentage of EP in cranio-spinal axis.

Ecchordosis physaliphora (EP) is a benign notochordal remnant frequently found at the retroclival prepontine cistern, but can occur anywhere between the sacrum and the base of the skull, often intradurally (Figure 1A–C,F). EP is an exceedingly rare and intriguing entity of the central nervous system (CNS), characterized by its distinct histological features and location. This enigmatic lesion, often mistaken for other, more common pathologies, has garnered considerable interest among clinicians and researchers.

**Figure 2:**
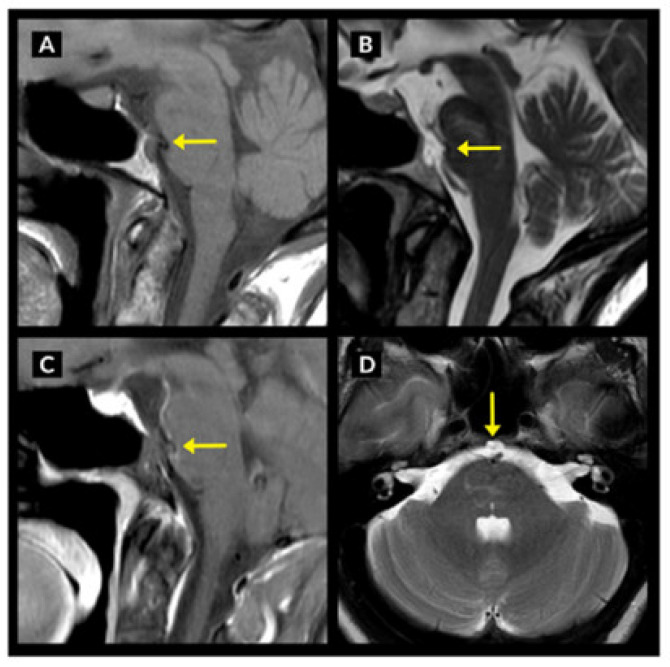
Brain showing a small T1 hypointense (**A**), T2 hyperintense (**B**,**D**) and non-enhancing (**C**) clival lesion extending into the prepontine cistern, displacing the basilar artery and mildly compressing the anterior surface of the pons (Lesion marked by yellow arrows).

EP has been found in 0.5–2% of all autopsies and 1.5% of all brain magnetic resonance imaging (MRI) images [1,2]. Figure 2 shows the MRI appearance of clival ecchordosis physaliphora extending into the pre-pontine cistern displacing the basilar artery and compressing the anterior surface of the pons.

It is important to differentiate EP from the chordoma. Chordomas are malignant neoplasms with notochordal differentiation most commonly on the axial skeleton requiring surgical management. In contrast to a chordoma, an EP is often asymptomatic and has a low proliferative index. Debates persist regarding the presence of contrast enhancementwithin EP and how it relates to its involvement with the clivus mainly because various studies provide inconsistent data relating to such points. The shared resemblance of both histopathological patterns between EP and more aggressive forms of chordoma alongside similar imaging observations brings up suggestions that may imply EP’s role serving as a precursor for chordoma; practices have unfolded since limited studies do not fully substantiate them. Differentiating EP from other pathologies is crucial for appropriate management.

The most common presenting clinical features of EP are headache, CSF rhinorrhea, diplopia, CN VI palsy and hemiparesis [3,4]. Less frequent symptoms include dizziness, tinnitus, facial pain, hearing loss, hemihypoesthesia, paresthesia and sudden death. Neuroimaging in combination with histopathologic and immunohistochemical characteristics can be helpful in making a diagnosis [3,4]. Neuroimaging remains the most useful diagnostic tool and guides whether to pursue conservative management with serial MRIs or surgical resection with biopsy [5,6]. A diverse clinical picture of EP can easily be muddled by other neurological ailments. The atypical presentation can be difficult to recognize, and, with higher-grade EP, can convert into malignant chordoma, which will require surgical management [3].

EP is a rare entity within theliterature, limited to few case reports and series. Due to its rarity, there is limited consensus regarding its clinical presentation, diagnostic workup and management strategies. Conducting a systematic review allows for a comprehensive and structured evaluation of the literature, enabling a robust understanding of EP, as no previous review is available in the literature. In this systematic review, we have attempted to analyze all the published symptomatic cases of EP until May 2023. By pooling and critically appraising the existing evidence, we seek to shed light on its epidemiology, clinical presentation, radiological findings, treatment strategies and overall outcomes. Furthermore, this review aims to identify gaps in knowledge, potential diagnostic challenges and future directions for research in the field of EP.

## 2. Methods

### 2.1. Study Protocol and Search Strategy

**Figure 3:**
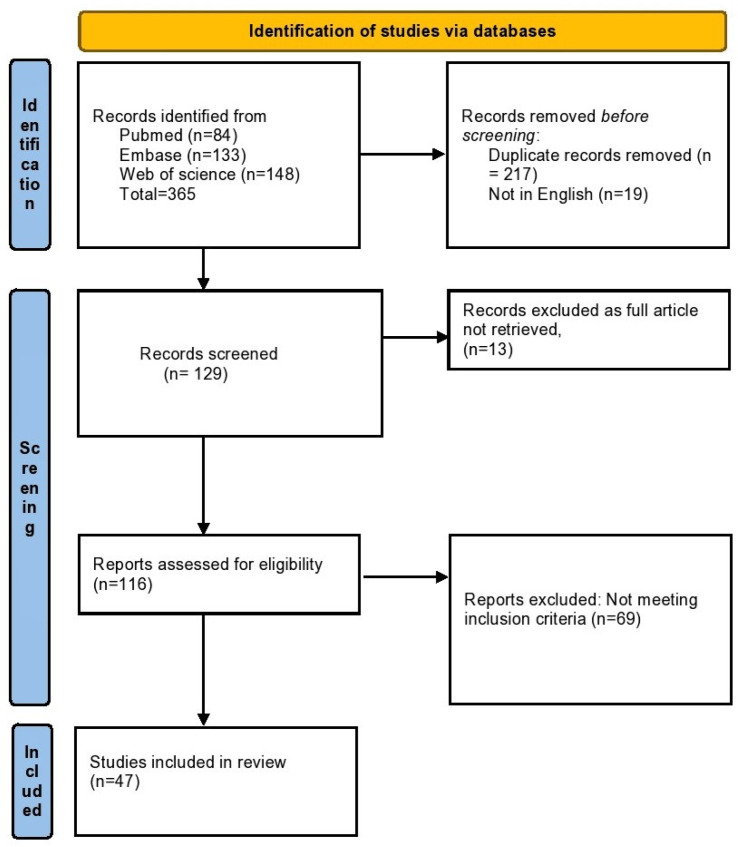
Flow diagram of the selection process.

We registered this systematic review with international database PROSPERO (registration number: CRD42022362189). A bibliographical search of PubMed, Web of Science and EMBASE was conducted for all studies published from 1 January 1982 to 31 May 2023, using the following search terms: (“ecchordosis physaliphora”). The reference list of included articles and reviews was searched for any remaining cases (Figure 3).

### 2.2. Study Selection Process

Inclusion criteria included (1) cases with MRI or histopathological evidence of EP and (2) patients presenting with neurological symptoms including headache, CSF rhinorrhea, diplopia, sensory loss, weakness, etc.

Exclusion criteria: included an incidental finding of unclear significance or asymptomatic cases, cadaveric studies, articles not having individual patient details, articles not retrieved, articles found in the non-English literature and articles re-publishing a previously reported case.

### 2.3. Quality Assessment and Data Acquisition

The overall quality of cases in case reports and case series was assessed using critical appraisal checklist for case reports provided by the Joanna Briggs Institute. Each article was carefully evaluated and read independently by TAR, AG and RKG, and systematically reviewed to identify additional cases in the reference list, which revealed an additional 3 cases. Demographic details, clinical symptoms, location of EP, management and outcomes were recorded and analyzed. Patients of all age groups were included and their age was recorded in years. Location of EP was grouped as prepontine (cystic mass in prepontine space/cistern), posterior sphenoid sinus wall (cystic mass in the sphenoid cavity or attached to its posterior wall), interpeduncular, C2 or extradural location, odontoid, intervertebral foramen and Dorello’s canal. Management was represented as conservative (no surgical intervention performed) and surgical. Outcomes were categorized as NED (no evidence of disease after surgical management), SF (symptom-free, clinically silent residual part of EP after a surgical procedure or conservative management), RD (residual disease, having mild persisting symptoms), SR (Symptom Recurrence after remission), ND (not defined) and death. Descriptive analysis wasperformedusing Microsoft Excel.

## 3. Results

This initial search yielded 365 results (84 from PubMed,133 from EMBASE and 148 from Web of Science). A total of 236 articles were removed for duplicates or not being in English. We later screened 129 articles for their abstracts and title, reviewed 116 full-text articles with respect to our inclusion and exclusion criteria, and finally included 47 studies and 59 cases in our review (Figure 3). After including our case of Reddy et al. [7], we analyzed a total of 60 cases (Table 1).

The average age of the patients was 46 years (median = 46, IQR = 18). The majority of patients were female (62%). Of the 60 cases in the review, the presenting symptoms, from most common to least common, were the following: headache (55%), CSF rhinorrhea (32%), diplopia (18%), CN VI palsy (15%), hemiparesis (8%), dizziness (5%), tinnitus (5%), facial pain (3%), hearing loss (3%), hemihypoesthesia (3%), confusion (3%) and paresthesia (4%) (Table 2). There were also single cases of back pain, weakness, gait disturbance, hyperalgesia, mutism, otalgia, thermohypesthesia, facial numbness and tremor. The presence of headache was more common in females (67%), while a few symptoms like facial pain and hearing loss were only present in women. Out of nine patients having CN VI palsy, eight patients had EP located in the prepontine region while one had it in Dorello’s canal.

The majority (77%) of the symptomatic EP lesions were in the prepontine region but a large minority (17%) were also found in the posterior wall of the sphenoid sinus. The majority of cases in the posterior sphenoid sinus wall were females (90%) (Table 3 and Figure 1E).

Forty-five (75%) of symptomatic cases were managed via surgical resection rather than conservatively (17%) via exclusively non-surgical interventions (e.g., serial MRIs, steroids, mannitol) (Table 4).

Of the surgical patients who were reported for outcomes (NA = 6) and received follow-up, 92% (*n* = 39) were either symptom-free or had no evidence of disease on imaging. In the reported literature, all cases of EP located at the posterior SS wall were managed surgically, whereas 80% of cases either showed no evidence of disease or were symptom-free.

## 4. Discussion

Ecchordosis physaliphora usually asymptomatic and is found during autopsy studies or an incidental radiological finding. The presence of pathologic ectopic notochordal tissue in the posterior clivus was first described by Luschka [51] in 1856. A year later, Virchow [52] depicted the first microscopic picture and called it Ecchondrosis physaliphora, believing it to be degenerative cartilage as a result of a process affecting spheno-occipital synchondrosis. Later, Muller [53] described its notochordal origin. On histological studies, Ribbert [54] confirmed its notochordal origin and coined the term ecchordosis physaliphora [35].

### 4.1. Classification

Some recent classifications are attempting to classify ecchordosis physaliphora. Lagman et al. [3] proposed a classification based on symptomatology, and radiologic features including size (6 cm^3^), gadolinium enhancement and bony erosion. EP is classified from grading I to V with grades IV and V having gadolinium enhancement and bony erosion representing malignant-transformation-warranted surgical resection to prevent further complication. Chiara et al. [55] also tried to classify EP based on imaging features using the FIESTA technique. Lesions were classified into classical EP, which shows T2 hyperintense excrescence (cyst-like composition) on the dorsal surface of the clivus, and possible EP. Classical EP was further classified into Type A (hyperintense excrescence on the dorsal surface of the clivus) and Type B (hyperintense excrescence with a hyperintense lesion within the clivus). Possible EP was further classified into incomplete EP (EP bud), hypointense protrusion of the clivus in T2; and EP variants, characterized by the hyperintense lesion(s) within the clivus alone.

### 4.2. Common Presentations

Our systematic review of symptomatic EP, the largest one conducted to date, finds that headache and CSF rhinorrhea are the two most common manifestations of symptomatic EPs. Also, 82% of the patients who had headaches due to meningitis (*N* = 11) presented with CSF rhinorrhea, which is relevant. Primitive notochord remnants sometimes perforate the dorsal clivus and reach into the intradural space called intradural EP, where they are connected with a thin bony stalk. The presence of EP on the clival surface can cause a mass effect leading to bony erosion and transclival fistula, or CSF fistula resulting in CSF rhinorrhea [1,11,15,16]. A majority of symptomatic patients had moderate symptoms and required surgical resections and repair of the fistula. In contrast, the literature suggests that asymptomatic EP usually warrants a conservative approach to management with a spaced follow-up to establish stability and avoid missing a rare, atypical chordoma presentation.

Other major clinical symptoms after headache and CSF rhinorrhea were diplopia and CN VI palsy found in 18% and 15% of patients, respectively. After leaving the brainstem, VI CN enters the prepontine region where it has a short tract, and later, after coursing through the two dural layers of clival dura, it enters into Dorello’s canal. From Dorello’s canal, the abducens nerve exits into the cavernous sinus [56]. During its course, the abducens nerve can becomeentangled or compressed by the mass arising in either the prepontine or Dorello’s canal region, leading to CN VI palsy and diplopia. This elucidates the ground basis of abducent palsy in only these spaces.

Our review found prepontine (77%) as the most common location and only one case with Dorello’s canal in symptomatic EP patients. Chihara et al. reported Dorello’s canal as the most common location for classical EP (82.4%). They did not have symptomatic EP patients as inclusion criteria for their study population, which could explain the reason for this discrepancy in the results.

### 4.3. Diagnosis of EP

#### 4.3.1. Imaging

There are no definite diagnostic criteria for EP, and diagnosis is usually made based on imaging characteristics. Typical EP lesions are cystic T2 hyperintensities, a T1 hypointensity with no contrast enhancement in the midline craniospinal axis along the dorsal aspect of the posterior clivus, are often at the level of Dorello’s canal [22], areless than six centimeters and have a stalk [3]. Atypical features of EP include an absent bony stalk, a T2 hypointense protrusion from the clivus, T2 hypointensities bordering the lesion, a T2 hypointense center within the lesion and a T2 hyperintensity on the pharyngeal surface or dorsum sellae [22].

#### 4.3.2. Immunohistochemical and Histopathological Analysis

In addition to imaging, immunohistochemical and histopathological analysis can help narrow the differential diagnosis. Positive immunohistochemical staining for cytokeratins AE1 and AE3, epithelial membrane antigen (EMA), S-100, galectin-3 and brachyury as well as negative staining for glial fibrillary acidic protein (GFAP), carcinoembryonic antigen (CEA) and 5′-nucleotidase suggest a notochord lesion but do not provide clarity in differentiating between EP and chordoma [4]. Histopathologic factors such as the presence of physaliphorous cells withlarge mucin-containing intracytoplasmic vacuoles, hypocellularity, lack of mitoses or necrosis, and sparse pleomorphism are signs of EP [4].

#### 4.3.3. Management and Outcomes

Data from the literature showed that surgical procedures were explored on 82% (*N* = 55) of the patients for definitive management and they revealed positive results where 92% (*N* = 39) of them either manifested no evidence of disease or were disease-free. The endoscopic endonasal transsphenoidal transclivalapproach (EEA) is the preferred surgical technique for addressing EP [20,24,46] as illustrated by Notaris et al. [57]. The EEA traverses natural routes to access the prepontine and retroclival region and gives the surgeon more room to visualize the brain stem and is also associated with less hospitalization and brain manipulation. The EEA can be adopted for fixing clival defects and CSF leaks along with EP resection and contingent reconstruction.

Early studies mentioned a surgical approach throughsuboccipital craniotomy [36,44,47], retrolabyrinthrinepresigmoid [28,37] and transmaxillary [12], which were associated with higher morbidities, complications and increased hospital stays. Few studies stuck to conservative management with steroids for acute states where patients presented with diplopia and VI CN palsy due to compression from EP and have shown satisfactory results [5,22]. Alkan et al. conservatively treated with osmotic diuretic for brain stem edema after intratumoral hemorrhage, causing a mass effect on the pons. Therefore, the use of conservative management was only implied in the acute compressive or edematous state but surgical removal is the preferred line of management.

#### 4.3.4. Differential Diagnoses

Chordoma(s) and EP are derived from common notochord remnants. It is difficult to differentiate them on the basis of their structure, histology and immunohistochemistry [58,59,60]. EP is a congenital benign malformation of the notochord in contrast to chordoma, which is a malignant transformation [61,62]. They are primarily extradural and cause bony erosion with respect to EP, which is commonly intracranial and intradural. EP is usually asymptomatic, while chordomas present with headache and multiple cranial nerve palsies as they grow, due to the mass effect on the brainstem [60,62]. They arerapidly progressive with poor prognosis even after surgical debulking and radiotherapy. On the CT scan, a tiny bony stalk connecting EP intradurally to the posterior clival surface has been noted, which is somewhat characteristic of EP [44,63]. MR imaging of chordomas [63] shows gadolinium contrast enhancement as a result of increased vascularity along with T1 and T2 hyperintensities and intratumoral calcification. Histologically, they demonstrate hypercellularity with cellular atypia and a higher Ki-67 index. A value of >5% in the MIB-1 index may corroborate the diagnosis of chordoma.

Benign notochordal cell tumors (BNCTs) share indistinguishable radiological findings with EP in contrast to chordoma. The existence of distributed physaliphorous cells with the absence of intercellular and extracellular matrices, positive immunohistochemistry for S-100 protein, vimentin, EMA and cytokeratin could aid in their diagnosis. Clinically, they are found in the midline of the clivus with no bony erosion [44,64].CLIPPERS also shows a T2 hyperintensity in the pontine region with gadolinium enhancement and a perivascular distribution. CSF analysis can be noted for leukocytosis, lymphocytosis, oligoclonal bands or glucose abnormalities, which are not in the case of EP.

#### 4.3.5. Limitations

This study may have inherent limitations of systematic review and it is possible that some case reports and case series were not included given the lack of description of cases. In some studies, patients were not followed up, or data regarding their management and outcomes were not recorded. Although these limitations have impacted this paper, the review is still significant in highlighting varied clinical presentations of EP and emphasizing the importance of considering EP in the differential diagnosis, especially in patients presenting with chronic symptoms suggestive of brainstem pathology and a retroclival or prepontine hyperintensity. Future directions based on large-scale multi-center studies focusing on the response of symptomatic EP patients to steroids could provide valuable insights into the efficacy of this treatment approach and help establish standardized management protocols. Exploring radiologically advanced techniques, such as Magnetic Resonance Spectroscopy (MRS), which provide metabolic details about the lesion can further enhance the accuracy of EP diagnosis and aid in distinguishing it from other lesions with similar imaging characteristics.

## 5. Conclusions

Based on our review of 60 cases published in the literature, we concluded that EP is more common in females (62%) with prepontine (77%) as the most common location. Although EP often presents asymptomatically, it is important to consider EP as a differential diagnosis when a patient presents with neurologic symptoms such as headache, CSF rhinorrhea or diplopia, especially in the setting of prepontine or posterior sphenoid sinus cystic lesions. It is typically visualized as cystic T2 hyperintensities with T1 hypointensities without contrast enhancement on MRI. While surgery is often the management of choice for symptomatic EP with 92% patients having favorable outcomes, preferably through the EEA, corticosteroids can be used for acute decompression especially in the setting of acute onset diplopia. In the context of differentiating between EP and chordoma, the extradural location and contrast enhancement are indeed important distinguishing factors.

## Figures and Tables

**Table 1:** Summary of ecchordosis physaliphora (EP) cases included in the systematic review.

Authors	Sex	Age	Symptom and Clinical Findings at Presentation(s)	EP Location	Management	Outcome
Adib et al. 2016 [8]	M	57	CN VI palsy, Diplopia, paresthesia	Prepontine	Surgical	RD
Ahn et al. 2016 [5]	M	15	CN VI palsy, diplopia	Prepontine	Conservative	SR
Akimoto et al. [9] 1996	F	51	Diplopia, headache	Prepontine	Surgical	ND
Alkan et al. 2009 [10]	M	22	Confusion, headache	Prepontine	Conservative	SF
Alli et al. 2008 [11]	F	52	CSF-L	Posterior SS wall	Surgical	NED
Bolzoni-Villaret et al. 2014 [6] #A	F	51	CSF-L	Posterior SS wall	Surgical	NED
#B	F	39	Diplopia, CN VI palsy	Prepontine	Surgical	NED
Cha et al. 2002 [12]	M	49	Dizziness, headache	Prepontine	Surgical	NED
Choudhri et al. 2014 [13]	M	63	Headache, tremor	Prepontine	Surgical	NED
Derakhshani et al. 2020 [14]	F	68	CSF-L, headache	Posterior SS wall	Surgical	NED
Dias et al. 2014 [15]	F	54	CSF-L, headache *	Posterior SS wall	Surgical	NED
Ferguson et al. 2016 [16]	F	ND	CSF-L, headache *	Prepontine	Surgical	ND
Filis et al. 2016 [17]	F	44	Headache	Prepontine	Surgical	NED
Fracasso et al. 2008 [18]	F	48	Sudden death **	Prepontine	Conservative	Death
Galloway et al. 2017 [19]	F	40	CSF-L, headache *	Posterior SS wall	Surgical	NED
Georgalas et al. 2020 [20] #A	M	81	CSF-L, headache *	Prepontine	Surgical	SF
#B	M	60	CSF-L, headache *	Posterior SS wall	Surgical	SF
#C	F	64	Headache *	Posterior SS wall	Surgical	SF
Ghimire et al. 2020 [21]	F	65	Hemiparesis, mutism	Prepontine	Surgical	NED
Ilorah et al. 2017 [22]	M	42	CN VI palsy, diplopia	Prepontine	Conservative	SF
Indiran V 2022 [23]	F	44	Headache	Prepontine	NA	NA
Kaul et al. 2013 [24]	F	52	Headache *, otalgia, tinnitus	Posterior SS wall	Surgical	SF
Krisht et al. 2013 [25]	F	17	Diplopia, headache CN VI palsy	Prepontine	Surgical	RD
Kurokawa et al. 1988 [26]	M	84	Hemiparesis, hyperalgesia, thermohypesthesia	C2, extradura	Surgical	ND
Lakhani et al. 2021 [27]	M	30	Headache	Prepontine	Conservative	SF
Ling et al. 2007 [28]	F	45	Hearing loss, tinnitus	Prepontine	Surgical	NED
LN Ang et al. 2020 [29]	F	43	Headache	Prepontine	Surgical	SF
MacDonald et al. 1990 [1]	F	66	CSF-L	Prepontine	Surgical	NED
Margo G et al. 2023 [30] #A	F	61	Headache	Prepontine	Conservative	SF
#B	M	41	Headache	Prepontine	Conservative	SF
Miki et al. 2017 [31]	F	44	Facial pain	Prepontine	Surgical	NED
Miki et al. 2008 [32]	M	59	Dizziness, gait disturbance	Prepontine	Surgical	NED
Ng et al. 1998 [33]	M	ND	Hemihypoesthesia, Hemiparesis	Odontoid process	ND	ND
Raffa A 2022 [34]	M	7	Headache	Prepontine	Surgical	SF
Reddy et al. 2022 [7]	F	55	Headache, paresthesia, weakness	Prepontine	Conservative	RD
Rengachary et al. 1997 [35]	F	34	Interscapular back pain	T8-T9 intervertebral foramen	Surgical	SF
Rodriguez et al. 1999 [36]	F	54	Dizziness	Prepontine	Surgical	ND
Rotondo et al. 2007 [37]	F	47	Facial pain, headache	Prepontine	Surgical	NED
Ruiz Castello MJ et al. 2023 [38]	F	46	Headache *, CSF L	Posterior SS wall	Surgical	NA
Sarkar N et al. 2022 [39]	M	16	Headache	Prepontine	Conservative	SF
Sooltangos et al. 2021 [40] #A	F	65	Hemiparesis, Confusion CSF L	Prepontine	Surgical	NED
#B	F	39	Headache, CSF L	Prepontine	Surgical	SF
#C	F	43	Headache *, CSF L	Prepontine	Surgical	NED
#D	F	39	Headache *, CSF L	Prepontine	Surgical	NED
#E	F	45	CSF L	Prepontine	Surgical	NA
Srinivasan et al. 2008 [41]	F	31	Headache	Prepontine	ND	ND
Stam et al. 1982 [2]	M	75	Sudden death **	Prepontine	ND	Death
Sun et al. 2020 [42]	F	22	CN VI palsy, Diplopia headache	Prepontine	Surgical	NED
Takeyama et al. 2006 [43]	M	12	Diplopia, hemiparesis	Prepontine	Surgical	NED
Toda et al. 1998 [44]	F	56	Headache	Prepontine	Surgical	NED
Touska et al. 2014 [45]	M	ND	Tinnitus	Prepontine	Conservative	ND
Veiceschi et al. 2021 [46] #A	M	59	CSF-L, headache *	Prepontine	Surgical	NED
#B	F	64	CSF-L	Prepontine	Surgical	NED
#C	M	41	CSF-L	Prepontine	Surgical	NED
#D	F	39	CN VI palsy, diplopia	Prepontine	Surgical	NED
#E	M	57	CSF-L	Prepontine	Surgical	NED
Watanabe et al. 1994 [47]	F	51	Hearing loss, facial hemihypoesthesia	Posterior SS wall	Surgical	RD
Wells et al. 2010 [48]	F	46	Headache, Facial numbness	Prepontine	NA	NA
Yamamoto et al. 2013 [49]	M	20	CN VI palsy, Diplopia	Dorello’s canal	Surgical	SF
Zhong et al. 2015 [50]	M	34	Diplopia, headache, CN VI palsy	Prepontine	Surgical	SF

Abbreviations: F, female; M, male; NA, not available; NED, no evidence of disease; ND, not documented; RD, residual disease, SF, symptom-free; SR, symptom recurrence. * These studies indicated meningitis as the clinical presentation, but headache was extrapolated as the presenting symptom given that headache is the most common presenting symptom of meningitis. ** These patients were found to have subarachnoid hemorrhages.(#A to #E for multiple cases in a case series).

**Table 2:** Frequency of symptoms and clinical findings at presentation in the review of symptomatic EP.

Symptoms and Clinical Findings at Presentation	Frequency (Case Count)	Frequency (% of Patients) *
Headache	33	55
CSF rhinorrhea	19	32
Diplopia	11	18
CN VI palsy	9	15
Other **	8	13
Hemiparesis	5	8
Dizziness	3	5
Tinnitus	3	5
Facial pain	2	3
Hearing loss	2	3
Hemihypoesthesia	2	3
Paresthesias	2	3
Confusion	2	3

* Many patients presented with multiple symptoms, so the sum of the above percentages of patients presenting with each symptom does not add up to 100%. ** includes single cases (2% of patients) of each of the following presenting symptoms: back pain, gait disturbance, hyperalgesia, mutism, otalgia, thermohypesthesia, facial numbness and tremor.

**Table 3:** Sex in relation to the location of EP.

Sex	Location of EP			Total
	Prepontine	Posterior Sphenoidal Sinus Wall	Others *	
Male	19	1	3	23
Female	27	9	1	37
Total	46	10	4	60

* Represents4 cases having a location at C2 andextradural, odontoid process, T8-T9 intervertebral foramen and Dorello’s canal.

**Table 4:** Showing outcomes in relation to management.

Total	Outcomes						Total
	No Evidence of Disease	Symptom Free	Symptom Recurrence	Residual Disease	Not Documented	Death	
**Surgical**	26	10	0	3	6	0	45
**Conservative**	0	6	1	1	1	1	10
**Not defined**	0	0	0	0	4	1	5
**Total**	26	16	1	4	11	2	60

## Data Availability

The data supporting the findings of this study are available from the corresponding author upon reasonable request.
